# Improving the Protection of Step-Down Transformers by Utilizing Percentage Differential Protection and Scale-Dependent Intrinsic Entropy

**DOI:** 10.3390/e27040444

**Published:** 2025-04-20

**Authors:** Chia-Wei Huang, Chih-Chiang Fang, Wei-Tai Hsu, Chih-Chung Yang, Li-Ting Zhou

**Affiliations:** 1 Department of Electrical Engineering, Zhaoqing University, Zhaoqing 526061, China; 2018013014@zqu.edu.cn (C.-W.H.); 2018013018@zqu.edu.cn (W.-T.H.); 2019013033@zqu.edu.cn (C.-C.Y.); zhoult149925@hanslaser.com (L.-T.Z.); 2 School of Computer Science and Software, Zhaoqing University, Zhaoqing 526061, China

**Keywords:** percentage differential protection, fault diagnosis, power transformer, complexity

## Abstract

Transformer operations are susceptible to both internal and external faults. This study primarily employed software to construct a power system simulation model featuring a step-down transformer. The simulation model comprised three single-phase transformers with ten tap positions at the secondary coil to analyze internal faults. Additionally, ten fault positions between the power transformer and the load were considered for external fault analysis. The protection scheme incorporated percentage differential protection for both the power transformer and the transmission line, aiming to explore fault characteristics. To mitigate the protection device’s sensitivity issues, the scale-dependent intrinsic entropy method was utilized as a decision support system to minimize power system protection misoperations. The results indicated the effectiveness and practicality of the auxiliary method through comprehensive failure analysis.

## 1. Introduction

The power transformer stands as a cornerstone of the power system, facilitating the alteration of voltage levels and the transmission of electric energy. It holds a pivotal role in the system’s infrastructure, serving as the nexus for conversion, where its secure operation directly influences the system’s continuous and stable functionality. With modern iterations characterized by substantial capacity, a wide voltage range, elevated costs, and intricate structures, the ramifications of failure are profound. Not only do such failures trigger extensive disruptions, but they also necessitate prolonged and intricate maintenance procedures, invariably resulting in substantial economic losses. Any malfunction of the power transformer detrimentally impacts both the safe operation of the power system and the reliability of power supply. Consequently, the imperative lies in the implementation of transformer protection mechanisms endowed with swift responsiveness, high reliability, and acute sensitivity [1,2]. The safeguarding of power transformers occupies a central position within both industrial and academic discourse. Multiple protection relays are deployed to ensure the integrity of these critical assets, with voltage per Hertz protection, differential protection, thermal protection, and overcurrent protection emerging as standard protocols [3].

Faults within transformers are categorized into internal and external types. Internal faults encompass anomalies occurring within the transformer enclosure, such as interphase short-circuit faults, inter-turn short circuits, and winding-to-core short circuits, among others. Conversely, external faults transpire outside the transformer, often resulting in elevated currents that compromise insulation integrity, leading to irreversible damage [4,5].

Differential protection serves as the primary safeguard for distribution transformers, operating on the fundamental principle of comparing current magnitudes and phases. However, the escalating issue of harmonic pollution exacerbates abnormalities in current magnitude and phase within distribution transformers [6,7,8]. Such anomalies generate increased unbalanced currents, thereby impeding the efficacy of differential protection. Furthermore, single line-to-ground faults on the secondary windings of grounded wye–wye (step-down) transformers pose unique challenges due to minimal short-circuit currents in the absence of a grounded neutral point. Consequently, protection devices exhibit abnormal operation as unbalanced currents escalate. Thus, the pursuit of effective strategies to enhance the sensitivity of differential protection remains a focal point of scholarly inquiry. Presently, most single line-to-ground faults on these transformers are coupled with supplementary protection mechanisms to achieve desired protective outcomes [9,10], alongside the establishment of fault diagnosis mechanisms predicated on periodic signal assumptions, leveraging signal processing techniques within power system analysis [11,12].

In the event of a power system failure, the fault signal comprises numerous transient signals generated alongside the typical signal at the moment of fault occurrence. These transient signal components exhibit temporal variability influenced by factors such as fault location, transition resistance at the fault position, and operational conditions of the system. Such transient signals epitomize a prototypical non-stationary random process. Transient signals, which are temporary occurrences with clear beginnings and endings, are inherently examples of non-stationary random processes. This is because their fundamental statistical attributes, like the mean and variance, undergo substantial changes over time.

In contrast to stationary processes where statistical features remain constant, the very nature of a transient event signifies a period of altered behavior, making its statistical description dependent on time. The characteristics of the signal before, during, and after the transient event are fundamentally different, directly violating the conditions for stationarity and thus positioning transient signals as a prime illustration of processes whose statistical behavior evolves. Hence, rapid detection of the fault occurrence, precise analysis of the fault signal, accurate localization of the fault transient and disturbance moments, and ensuring timely action of the protection device emerge as pressing challenges. Recent research underscores that fault diagnosis predominantly encompasses two facets: the information source of fault diagnosis and the methodology employed for fault diagnosis. Initial scrutiny involves characterizing and analyzing the signal under study within the context of power system signal processing. Subsequently, it entails delineating the parameters to be measured and the requisite level of measurement accuracy [13,14].

Fault signals acquired by measuring devices in power systems frequently exhibit nonlinear and non-stationary characteristics. These fault features often confront attenuation and interference from robust ambient noise and adjacent components. However, prevailing methodologies largely rely on a stationary assumption, proving inefficient in analyzing these intricate fault signals. Consequently, the adoption of entropy-based statistical measures has gained traction, facilitating the quantification of complexity and detection of dynamic changes by accommodating the nonlinear dynamics of time series. Notably, entropy-based approaches have witnessed widespread adoption in power system fault diagnosis [15].

In contrast, conventional signal analysis often operates under the assumption of stationarity, implying that a signal’s statistical properties remain consistent over time. However, complex fault signals typically defy this assumption, exhibiting non-stationary behaviors such as abrupt shifts or intermittent patterns. Applying methods designed for stationary signals to these fault signals proves ineffective because such approaches are unable to capture the time-varying characteristics that are critical for accurate fault detection and diagnosis. The evolving statistical properties of fault signals directly contradict the fundamental principles of stationary analysis, leading to potentially inaccurate or incomplete interpretations and highlighting the need for techniques capable of effectively handling non-stationary data. In this context, scale-dependent intrinsic entropy emerges as a systematic approach leveraging the empirical mode decomposition (EMD) method. This approach enables the removal of time series across various time scales, thus facilitating the analysis of signal complexity by assessing dynamic irregularities across multiple time scales [16].

This study integrated percentage differential protection with the scale-dependent intrinsic entropy methodology for power transformer protection. The proposed methodology entails acquiring the measuring current and controlling the action or inaction of the protection device through percentage differential protection. Subsequent signal processing within power system analysis aims to ascertain the complexity of the fault signal, thereby aiding in the identification of abnormal operation and fault types, consequently enhancing the reliability and safety of relay protection. The simulation results highlight the efficacy and practicality of the combined approach proposed herein.

## 2. Method

### 2.1. Percentage Differential Protection

Percentage differential protection stands as the primary safeguard for transformers, offering distinct advantages such as safeguarding against phase failures, ground faults, and inter-turn short circuits within the transformer’s internal windings and leads. In practical application, differential relays are typically installed on both the primary and secondary sides of the transformer to monitor operational conditions. Adhering to the principle of energy conservation, the protection device is triggered once abnormal current monitoring on both sides surpasses the predetermined threshold, leading to the disconnection of the transformer from the power system [17,18]. In alignment with the transformer’s characteristics and the differential protection principle, the turns ratio of the current transformer (CT) deployed on the primary and secondary sides of the transformer is carefully selected. During normal operation and in the event of external faults, the currents on the secondary side of the CTs on both sides of the transformer are equal in magnitude and opposite in direction, ensuring a zero-differential current. For instance, in the case of a wye–wye connected transformer with a neutral wire on both sides, the following equation applies [19]:

(1) $$I_{H}^{\prime }=I_{L}^{\prime }=\frac{I_{H}}{n_{TA.H}}=\frac{I_{L}}{n_{TA.L}}\;\mathrm{or}\;\frac{n_{TA.L}}{n_{TA.H}}=\frac{I_{L}}{I_{H}}=n_{T}$$

where $n_{TA.H}$ denotes the turns of the primary coil; $n_{TA.L}$ denotes the turns of the secondary coil; $n_{T}$ denotes the turns ratio of the transformer; $I_{H}^{\prime }$: current of the secondary circuit on the low voltage side; $I_{L}^{\prime }$: current of the secondary circuit on the high voltage side. The implementation of percentage differential protection for transformers aims to mitigate unbalanced currents within the differential circuit. Unbalanced currents can arise from various sources, including excitation inrush currents—typically 6 to 8 times the rated current—as well as discrepancies between the actual and calculated turns ratios of the CTs, transformer tap adjustments, and symmetric and asymmetric faults [20]. With the exception of unbalanced currents resulting from symmetric and asymmetric faults, other sources can be addressed through signal analysis or numerical calculations. Consequently, this study focuses solely on unbalanced currents stemming from symmetric and asymmetric faults.

Digital filters are employed to eliminate aperiodic components, effectively mitigating the impact of unbalanced currents caused by symmetric and asymmetric faults. The underlying principle of percentage differential protection guides this approach. An empirical equation representing the maximum unbalanced current for percentage differential protection is expressed as follows [21]:

(2) $$I_{ub.\max}=\left(10\%K_{st}K_{aper}+\Delta U+\Delta m\right)I_{k.\max}/n_{TA}$$

where 10% is the maximum relative error allowed by the CT selected by the error curve; $K_{st}$ denotes the homogeneous coefficient of the CT; Δm represents the error arising from the non-complete matching of CT turns ratios after compensation, and inherent error in the microcomputer protection device; $K_{aper}$ denotes the aperiodic component coefficient of the CT; ∆U represents the relative error due to the voltage regulation of the on-load voltage regulating transformer.

Considering that unbalanced currents surge due to both symmetric and asymmetric faults, percentage differential protection is adopted. The differential current is determined based on the maximum unbalanced current necessary to avoid such faults, establishing action parameters based on the percentage differential characteristic. The following equation represents this principle [21]:

(3) $$I_{d}>K_{res}I_{res}+I_{d.\min}$$

where $I_{d}$ denotes the differential current; $I_{res}$ denotes the restrained current; $I_{d.min}$ denotes the starting current, also known as the minimum differential current; $K_{res}$ denotes the slope based on the percentage restrain characteristic, denoted as the restrain coefficient, with $K_{res}=\tan\alpha$. The two-polyline characteristic can be represented by the following action equations [21].

(4) $$\left\{ \begin{array}{ll} I_{d}>I_{d.\min} & \;\mathrm{if}\;I_{res}<I_{res1}\\ I_{d}>K\left(I_{res}-I_{res1}\right)+I_{d.\min} & \;\mathrm{if}\;I_{res}\geq I_{res1} \end{array}\right.$$

where $I_{res1}$ denotes the inflection restrain current. During regular operation, the unbalanced differential current of the transformer remains minimal. The minimum differential current for percentage differential protection is established based on the unbalanced current necessitated to prevent the transformer from operating at maximum load current, $I_{L.max}$. Typically, in this case, $I_{d.min}=\left(0.2\sim 0.5\right)I_{n}$, the inflection restrain current, $I_{res1}$, slightly exceeds $I_{d.min}$, $I_{res1}=\left(0.6\sim 1.1\right)I_{n}$, where *I_n_* denotes the reference current, i.e., the rated secondary current of the reference side. The slope of the polyline based on the percentage restraint characteristic is defined as follows [21]:

(5) $$K_{res}=\left(K_{res}I_{ub.\max}-I_{d.\min}\right)/\left(I_{res.\max}-I_{res1}\right)$$

Percentage differential protection works by comparing the currents flowing into and out of a protected zone, which should ideally be identical during normal operation. In this scheme, filters play a vital role in enhancing performance by reducing the impact of less-than-ideal conditions. More specifically, filters can help prevent or restrain the relay from operating during events such as transformer inrush currents (which are rich in harmonics) or external faults that cause current transformer saturation (potentially leading to false differential currents). By selectively eliminating these undesirable components, the relay’s security (avoiding incorrect tripping) and dependability (correctly tripping for internal faults) are significantly improved, ensuring accurate and reliable protection of the power system equipment.

### 2.2. Scale-Dependent Intrinsic Entropy

Multiscale entropy (MSE), an extension of standard sample entropy measures, provides insights into the complexity of variations across a range of time scales. In essence, a coarse-grained scaling function of entropy illustrates the complexity of finite-length time series. The characterization of the time series for a coarse-grained process is defined as follows [22,23]:

(6) $$x^{\tau }(l)=\frac{1}{\tau }\sum_{t=(l-1)\tau +1}^{l\tau }x(t)$$

where $x^{\tau }\left(l\right)$ represents the coarse-grained time series with a coarse-grained scale τ; l denotes the new index of the coarse-grained time series and 1≤l≤N/τ; x(t) represents the original time series; N denotes the length of the actual time series; N/τ is the length of each coarse-grained time series.

EMD serves as an adaptive detrending algorithm for nonlinear and multimodal time series [24]. The complexity of the detrended time series is quantified by the MSE algorithm with an entropy set spanning coarse-grained scales from 1 to L. Each entropy is computed on a detrended coarse-grained time series [16,22]. When a specific nth intrinsic mode function (IMF) detrends the time series, the complexity contributed is considered a set of entropy increments on the coarse-grained scale of 1 to L. The entropy increment is defined as follows:

(7) $$\Delta E_{n}^{\tau }=E_{n}^{\tau }-E_{n-1}^{\tau }$$

The overall entropy distribution of the time series is represented by an entropy increment matrix of size *M × L*, where *M* represents the IMFs of the time series and *L* denotes the entropy increments. Since each IMF possesses its internal time scale, the entropy increase contributed by a specific IMF is meaningful only within a specific coarse-grained scale related to the IMF’s internal time scale. The system complexity of the time series is considered the set of internal entropy, with each individual internal entropy representing the entropy contributed by the specific IMF on its corresponding scale [23].

The average period of oscillation and fluctuation defines the time scale of IMF, as expressed in the following equation [25]:

(8) $${\bar{T}}_{n}=\int S_{\ln T,n}d\ln T{\left(\int S_{\ln T,n}\frac{d\ln T}{T}\right)}^{-1}$$

where $S_{\ln T,n}$ represents the Fourier spectrum of the nth IMF as a function of ln*T*; $\bar{T}$ denotes the period; represents the average period of the nth IMF. The intrinsic time scale is defined as the average period of the IMF to explore the correlation between the intrinsic time scale and the particular coarse-grained scale. 

**Figure 1:**
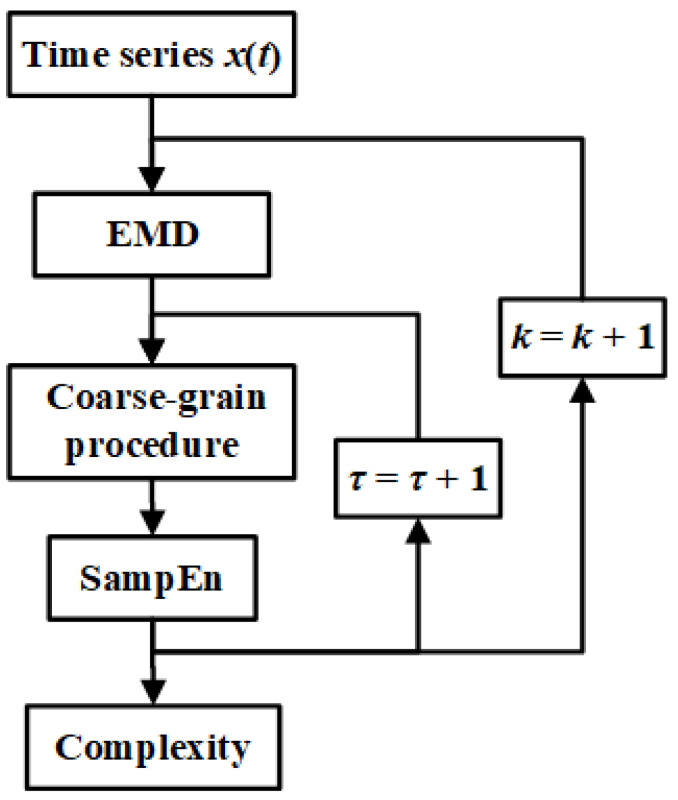
Flow chart of the scale-dependent intrinsic entropy.

Figure 1 illustrates the entire process employing the scale-dependent intrinsic entropy algorithm. Initially, the time series is decomposed into Intrinsic Mode Functions (IMFs) using the EMD method. At a given scale factor *τ*, the Sample Entropy (SampEn) of all coarse-grained time series at this scale factor are computed. The measure of complexity is determined by calculating the mean of all entropy values at the designated scale factor *τ*.

Scale-dependent intrinsic entropy involves calculating entropy across various scales of a time series, often using methods like EMD. For the resulting entropy matrix, the computational resources needed for implementation on an embedded platform are primarily determined by the matrix’s dimensions (number of scales versus the number of intrinsic mode functions or segments), the specific entropy measure employed (e.g., Shannon, Sample Entropy, Fuzzy Entropy), and the efficiency of the matrix operations. Generally, entropy calculation involves logarithmic and summation operations, which can be computationally demanding, mainly when performed repeatedly across a potentially large entropy matrix. Memory demands will also be influenced by the matrix size and the necessity of storing intermediate results, presenting challenges for embedded systems with limited resources, thus necessitating careful algorithm optimization and potentially hardware acceleration to achieve real-time performance.

## 3. Results

**Figure 2:**
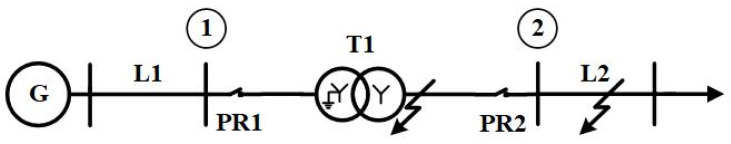
Schematic diagram.

A 50 Hz, 10,000 MVA, a 110 kV power source (G) is connected to bus #1 through a transmission line (L1=50 km, $Z_{1}=Z_{2}=0.17+j0.4\;\Omega /\mathrm{km}$). Subsequently, a step-down grounded wye–wye transformer (T1) is connected to bus #2, supplying a load (P = 1 MW, Q = 0.6 MVAR) via another transmission line (L2 = 20 km, $Z_{1}=Z_{2}=0.17+j0.4\;\Omega /\mathrm{km}$). The single-phase rating of the transformer is 20 MVA, 110/11 kV ($V_{s}\%=10.5$, $\Delta P_{s}=135\;\mathrm{kW}$, $I_{O}\%=0.8$, $\Delta P_{O}=22\;\mathrm{kW}$), with 10 tap positions on the secondary coil. The schematic diagram is illustrated in Figure 2. The simulation model is constructed using Simulink. Voltage and current signals of phases A, B, and C, sampled at $5\times 10^{-6}$, are utilized in the study. Current transformers (CTs) with circuit breakers are positioned at PR1 and PR2, with turns ratios of 1/40 and 1/400, respectively. Various types of faults between bus #1 and bus #2, including those on transmission line L2, are introduced to analyze internal and external fault characteristics. Voltage and current signals are measured at bus #1 and bus #2. The fault removal time of the protection device encompasses both the operation time of the protection device and the circuit breaker. Generally, fast protection operates within 0.04 s to 0.08 s, with the fastest protection reaching 0.01 s to 0.04 s. Tripping times for typical circuit breakers range from 0.06 s to 0.15 s, while the fastest protection achieves 0.02 s to 0.06 s [26]. Thus, the failure duration for this simulation model is set to 0.1 s. In a practical implementation, the sampling frequency for acquiring voltage and current data should be considerably higher than the highest frequency component of interest within the electrical network, typically ranging from several kilohertz to tens of kilohertz. This ensures accurate capture of transient events and harmonic content. The chosen sampling frequency has a critical impact on the performance of the proposed scheme. Insufficient sampling can result in aliasing, a distorted representation of fault characteristics, and delayed or incorrect protection responses. Conversely, excessively high sampling rates can increase the computational load without a proportional improvement in performance. Disturbances present in electrical networks, such as voltage sags, swells, harmonics, and high-frequency noise, can distort the measured voltage and current signals. If the proposed protection scheme is not designed to be resilient against these disturbances through appropriate filtering and signal processing, it could lead to incorrect operation or even failure. Setting an accurate threshold for triggering transformer protection requires careful consideration of the transformer’s operational characteristics, potential fault currents, inrush currents, and sympathetic inrush. This often involves a combination of instantaneous and time-delayed thresholds, techniques like harmonic restraint or blocking, and adaptive settings that adjust based on the transformer’s operating conditions. The goal is to ensure both high sensitivity to actual faults and robust security against unwanted tripping.

Three single-phase transformers are employed instead of three-phase transformers. In this single-phase transformer, users can adjust the number of primary and secondary windings as necessary, along with the coil tap for each winding. The secondary coil is divided into 10 equal parts, allowing for tap adjustments at 10%, 20%, 30%, 40%, 50%, 60%, 70%, 80%, 90%, and 100%, respectively.

**Figure 3:**
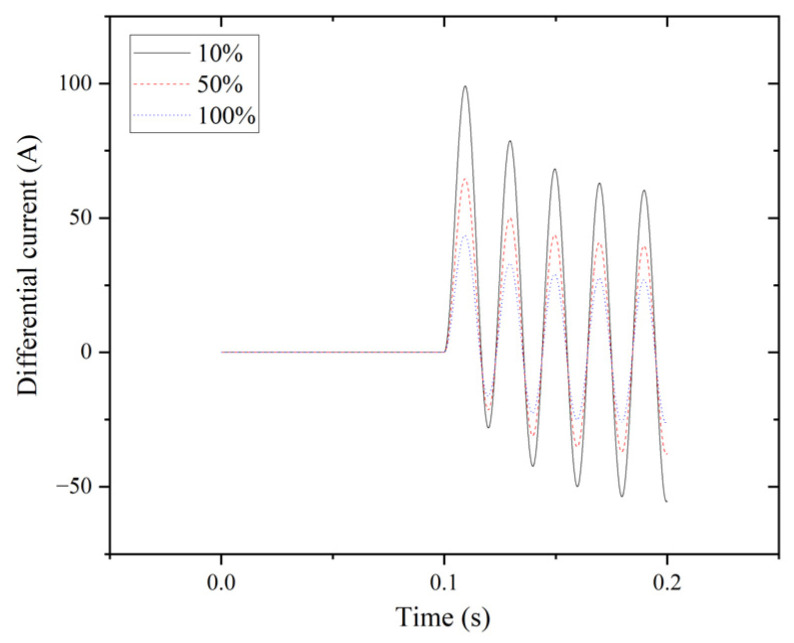
LLL fault for phase A.

Percentage differential protection is employed in the system to analyze internal fault characteristics. The simulation duration ranges from zero to 0.2 s. The results of the differential current at tap positions of 10%, 50%, and 100% for the triple line-to-line fault (LLL) are depicted in Figure 3. Transient generation is accompanied by fault occurrence, commencing at 0.1 s and concluding at 0.2 s.

**Figure 4:**
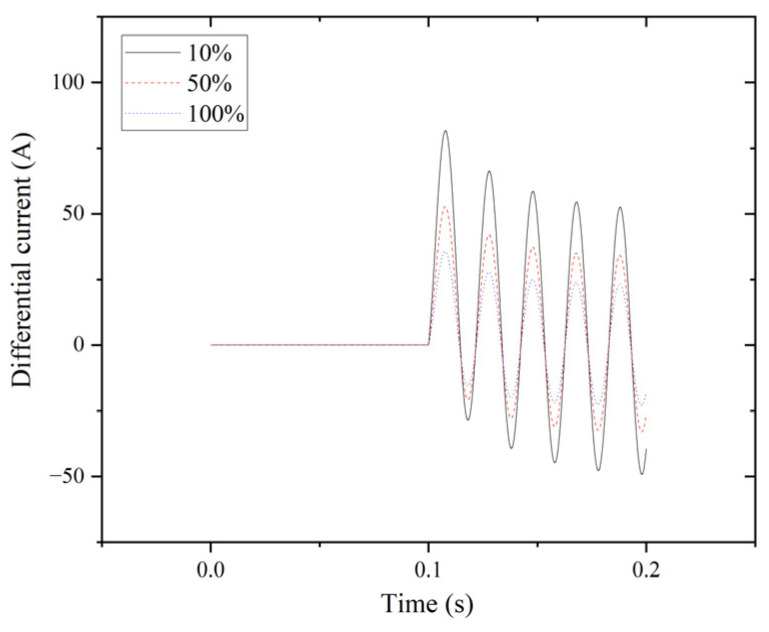
LLG fault for phase A.

The results of the differential current for the double line-to-ground fault (LLG) are illustrated in Figure 4.

**Figure 5:**
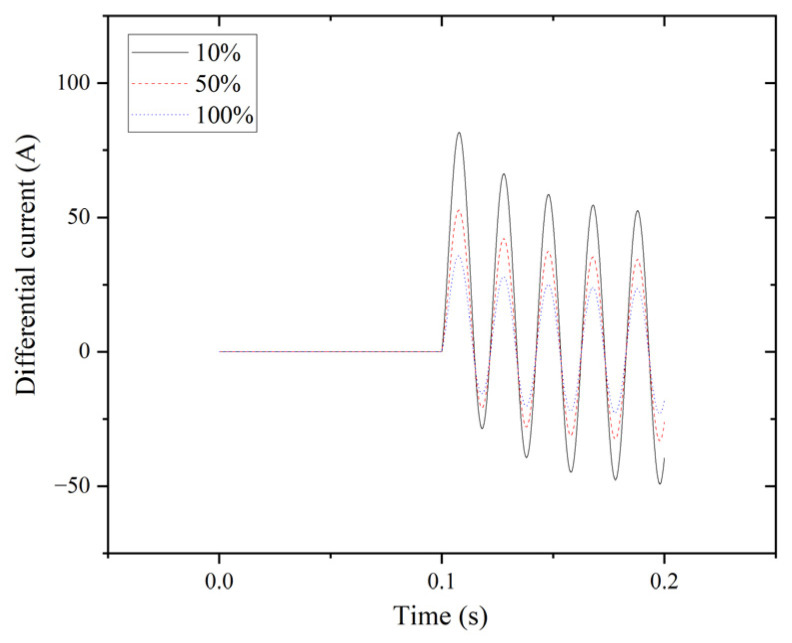
LL fault for phase A.

The differential current results for the double line-to-line fault (LL) are presented in Figure 5.

**Figure 6:**
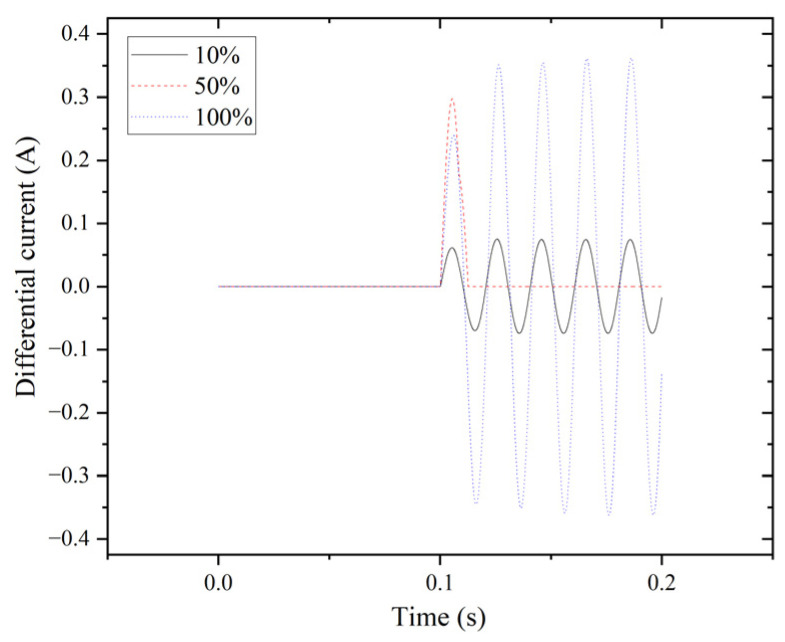
LG fault for phase A.

These results indicate that under LL, LLG, and LL faults, the greater the number of short-circuited turns, the higher the short-circuit current and subsequent differential current. The differential current results for the LG fault are displayed in Figure 6.

**Figure 7:**
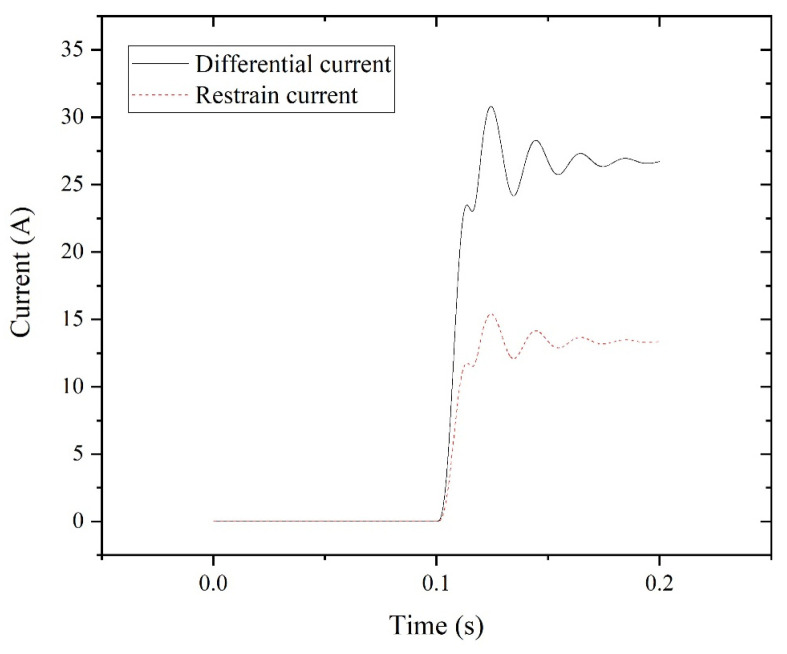
Differential and restraint currents for LL fault.

The simulated results of the differential current and restraint current, in absolute value at 100% for phase A when the LL fault occurs, are illustrated in Figure 7.

**Figure 8:**
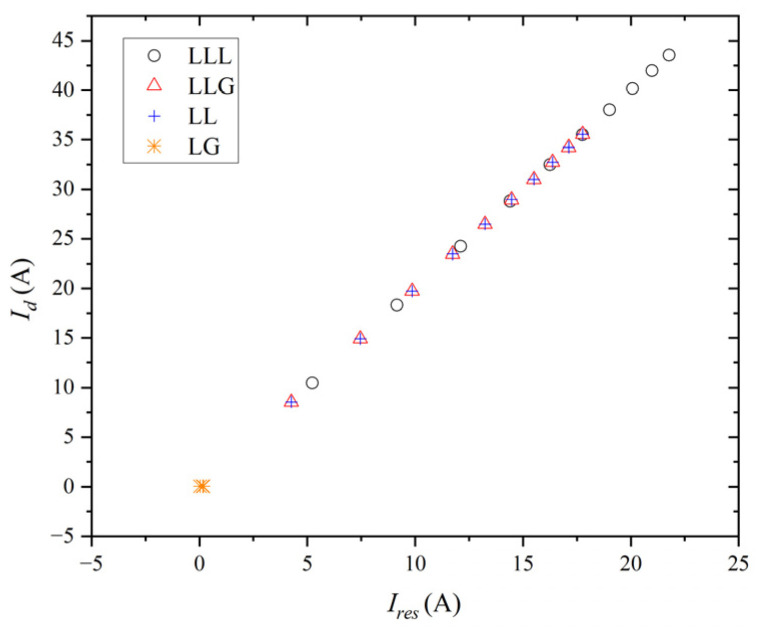
Operating diagram based on percentage differential protection for phase A of internal fault.

Figure 8 depicts the simulation results of the operating diagram based on percentage differential protection when symmetrical and unsymmetrical faults occur in the system. The tap position in Figure 8 ranges from the lower-left (10%) to the upper-right (100%).

As shown in Figure 8, the LG fault registers near zero due to the grounded wye–wye transformer, enhancing the protection device’s sensitivity. To address this issue, the scale-dependent intrinsic entropy method assists in diagnosing the LG fault from a signal perspective. The complexity results for the current at bus #2 are presented in Table 1. When the LG fault arises in the system, the complexity of the current for the fault phase increases, as does the complexity for the non-faulty phases. This complexity is higher compared to the normal operating condition for each phase.

As indicated by the data in Table 1, the short-circuit current of the LG fault increases with the number of short-circuited turns. Because the neutral point is not grounded, the short-circuit current of the LG fault is capacitive. Therefore, with fewer short-circuited turns, the equivalent value of the inductive reactance on the circuit is considerably high, leading to lower capacitive reactance. Consequently, the short-circuit current for the LG fault is relatively large. This result also confirms the phenomenon depicted in Figure 6, wherein fewer short-circuit turns correspond to a larger differential current. The complexity results for the voltage at bus #2 are provided in Table 2.

For analyzing turn-to-turn faults (TT), a practical scheme employs zero sequence voltage protection in the system [2]. For phase A, the fault module connects to any two individual tap positions to enable the LLG fault. Instead of utilizing zero sequence voltage protection, the scale-dependent intrinsic entropy method assists in diagnosing the TT fault. Table 3 displays the complexity of the current for the TT fault at bus #2. For example, when Tap#A position at 2.1 is connected to the Tap#B position at 2.5, the complexity is 1.4443. The TT fault in the system increases the complexity of the current for the fault phase.

The complexity of the voltage for the TT fault at bus #2 is depicted in Table 4. When the TT fault occurs, the voltage complexity for the fault phase decreases with increasing distance between two tap positions.

**Figure 9:**
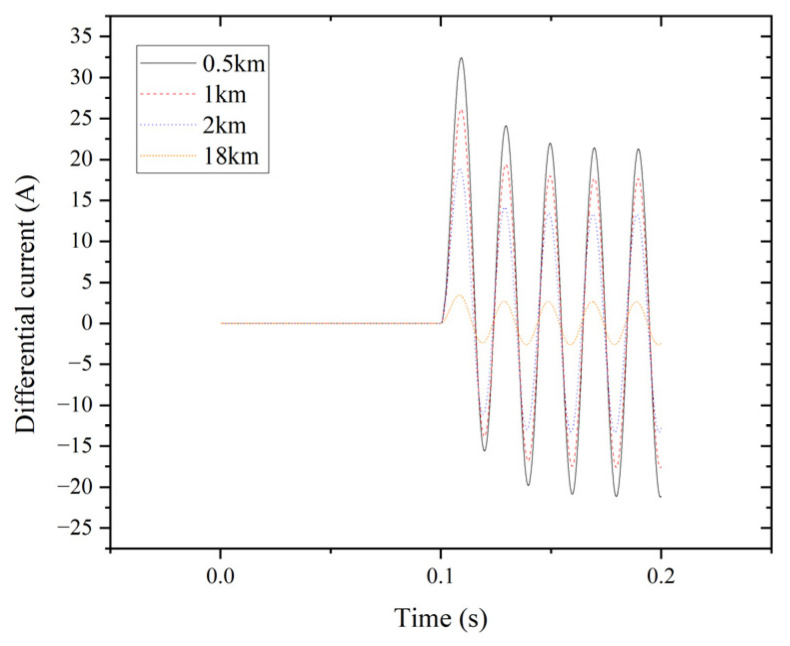
Differential current of phase A for the LLL fault.

The system employs percentage differential protection to analyze the characteristics of external faults. The transformer’s secondary connects to transmission line (L2), which spans 20 km, divided into ten segments of 2 km each. Adding two extra points, 0.5 km and 1 km, helps examine the edge between internal and external faults. Figure 9 illustrates the differential current for phase A at 0.5 km, 1 km, 2 km, and 18 km of the transmission line during an LLL fault.

**Figure 10:**
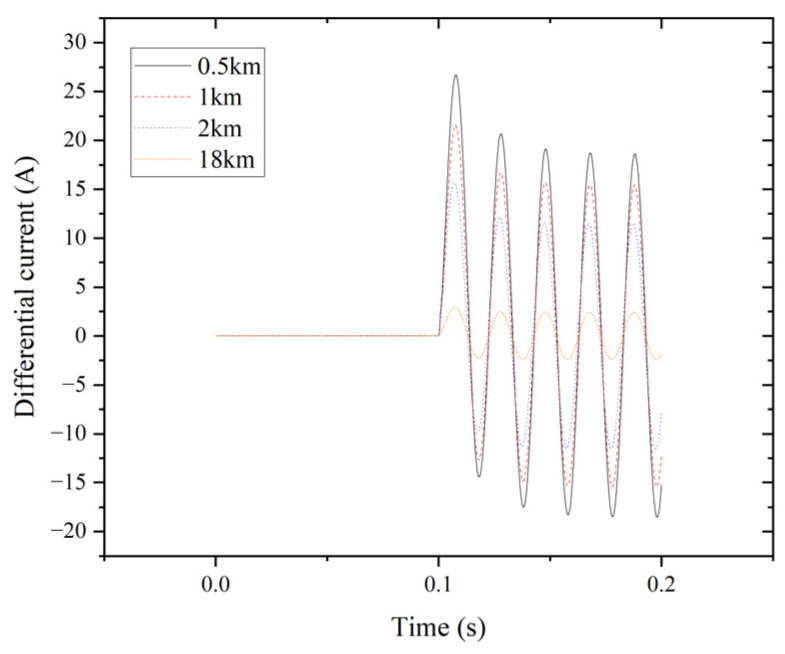
Differential current of the phase A of LLG fault.

Figure 10 depicts the differential current for phase A at 0.5 km, 1 km, 2 km, and 18 km of the transmission line during an LLG fault.

**Figure 11:**
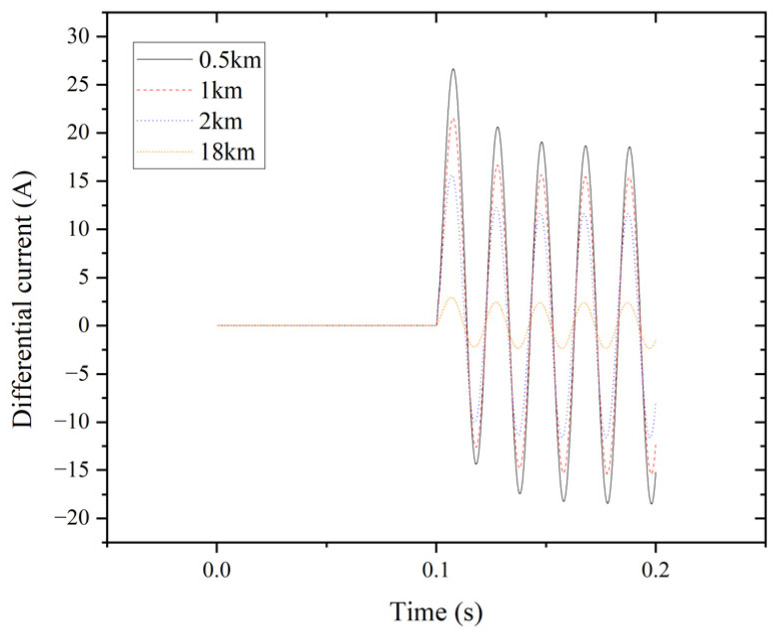
Differential current of the phase A of LL fault.

Figure 11 depicts the differential current for phase A at 0.5 km, 1 km, 2 km, and 18 km of the transmission line during an LL fault.

**Figure 12:**
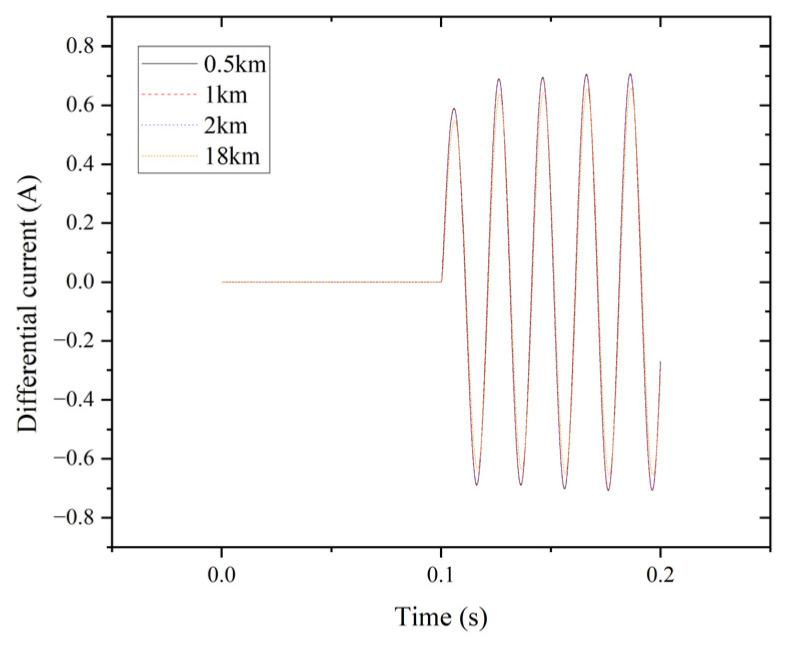
Differential current of phase A for the LG fault.

Figure 12 illustrates the differential currents for phase A at 0.5 km, 1 km, 2 km, and 18 km of the transmission line during an LG fault. The maximum differential current at the measurement point is minimal because of the small short-circuit current for the LG fault, accompanied by aperiodic noise.

**Figure 13:**
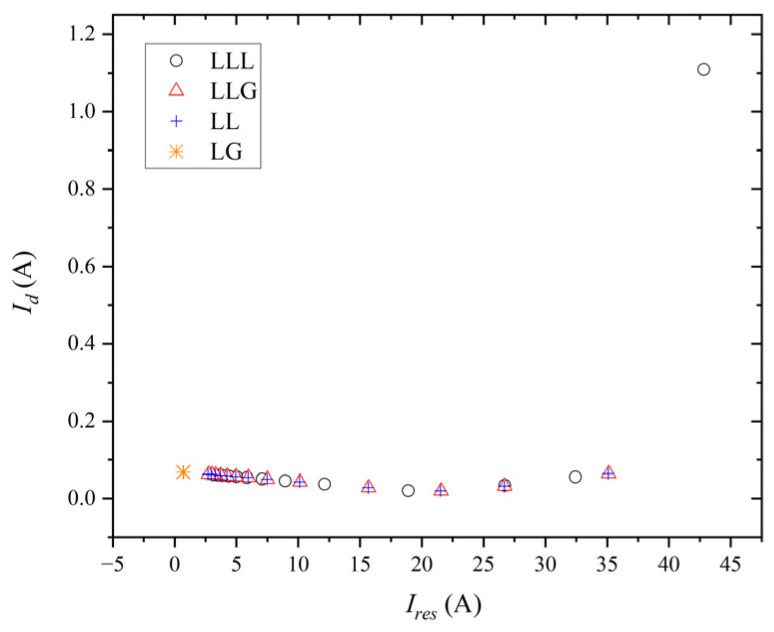
Operating diagram of percentage differential protection for phase A of the external fault.

Figure 13 displays the simulation results of the differential current versus the restraint current when symmetrical and unsymmetrical faults are applied in the system. The transmission line location in Figure 13 ranges from the lower-left (20 km) to the upper-right (0 km).

When the LG fault occurs externally, a similar scenario arises, as depicted in Figure 13. At certain distances, the differential current approaches zero, nearing the minimum operational threshold, resulting in the malfunction of the protection device. Employing the scale-dependent intrinsic entropy method aids in diagnosing the LG fault from a signal perspective. Table 5 illustrates the complexity of the current measured at bus #2. The occurrence of the LG fault in the system increases the current’s complexity for the fault phase, along with a rise in complexity for the non-faulty phases. This complexity surpasses that of the normal operating condition for each phase, both fault and non-faulty.

The complexity of the voltage measured at bus #2 is detailed in Table 6. With the occurrence of the LG fault in the system, the voltage complexity for the fault phase escalates. As distance increases, complexity also rises. Compared with regular operation, complexity exhibits a wider range of changes when a fault occurs. The complexity of the voltage for the non-faulty phases shows a similar trend but within a narrower range compared to the fault phase. Complexity increases for the non-faulty phases, indicating a deviation from their normal operating condition.

**Figure 14:**
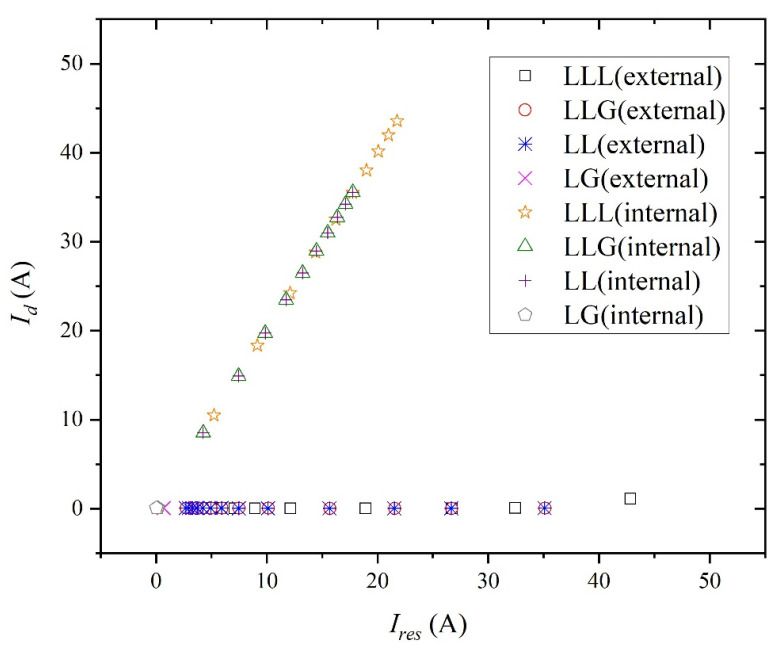
Operating diagram based on percentage differential protection for phase A of internal and external faults.

The combined simulation results encompass both internal and external faults, depicted in Figure 14. When discussing symmetrical and unsymmetrical faults, the minimum operating current deemed reliable is less than 0.0347 A, as outlined in this study. To prevent misoperation stemming from other factors, the minimum operating current is conservatively set to 0.028 A. However, this value is excessively low, leading to potential misoperations such as circuit breaker tripping due to current instability during system startup. Consequently, the application of the scale-dependent intrinsic entropy method is essential, particularly in addressing instances of the LG fault, without impeding the functionality of protective devices utilized for internal protection.

## 4. Discussion

Based on the aforementioned simulations, the percentage differential protection proves dependable for all faults except the LG and TT faults, attributed to the grounded wye–wye transformer configuration discussed in this study. To prevent malfunction, the minimum differential current must be lower than the minimum value of all fault currents. However, excessively low values may trigger protection device misoperation during normal operation, especially notable in the case of the highly sensitive protection device response to LG faults associated with grounded wye–wye transformers.

This paper advocates for the utilization of the scale-dependent intrinsic entropy method to aid in transformer fault diagnosis. By assessing the complexity of voltage and current signals at fault points, this method discerns fault occurrence, mitigating percentage differential protection malfunctions during different LG and TT fault scenarios. Addressing the challenge posed by excessively low minimum differential current settings in protection devices, the method distinguishes internal and external faults by analyzing fault phase voltage complexity, as shown in Table 2, Table 4, and Table 6. Both internal and TT faults are discerned based on the fault phase’s voltage complexity, yielding consistent conclusions across Table 2 and Table 4. The method’s efficacy in indicating the system’s current status—normal or faulty—through data analysis aids in determining faults or non-faults, as demonstrated by simulation results.

## 5. Conclusions

This study simulated a grounded wye–wye transformer under internal fault conditions across various tap positions and TT faults at the secondary coil for phase A. Additionally, symmetrical and unsymmetrical faults were explored at different distances for external faults. The simulation data, utilized for generating line charts and waveform diagrams, were derived based on the protection criterion set for the protection device, which operated effectively in response to various faults.

To address the limitations in diagnosing LG and TT faults, this study incorporated scale-dependent intrinsic entropy. The viability of this method was demonstrated by evaluating the complexity value of the power system, regardless of its standardization. Moreover, the proposed method distinguishes between internal and external faults, as well as internal and TT faults, based on the voltage complexity of the fault phase. Combining percentage differential protection with the scale-dependent intrinsic entropy method proved effective for transformer internal and external fault diagnosis. Overall, the proposed method enables online fault diagnosis, presenting as a feasible approach for power transformer protection through power system signal analysis. This paper can serve as a reference for actual power transformer protection fault diagnosis.

While the proposed approach offers a promising direction, a comprehensive comparison between simulation results and actual experimental data would substantially strengthen its validation and practical relevance. Such an analysis would reveal the accuracy and limitations of the simulation models in capturing the intricacies of a real-world system, highlighting any discrepancies that arise from unmodeled factors, sensor inaccuracies, or environmental effects. By quantifying these differences and pinpointing areas of agreement and divergence, the study could be enhanced by refining the simulation parameters or suggesting necessary adjustments for future real-world deployment, ultimately increasing the credibility and real-world applicability of the research.

## Figures and Tables

**Table 1:** Complexity of current for the LG fault.

Tap	Fault Phase A	Non-Faulty Phase B	Non-Faulty Phase C	Normal Phase A	Normal Phase B	Normal Phase C
10%	1.4661	1.5531	1.4039	1.0778	1.0694	1.0667
20%	1.4761	1.5631	1.3064	1.0778	1.0694	1.0667
30%	1.4875	1.5743	1.2236	1.0778	1.0694	1.0667
40%	1.5002	1.5788	1.1779	1.0778	1.0694	1.0667
50%	1.5152	1.5690	1.1471	1.0778	1.0694	1.0667
60%	1.5341	1.5409	1.1252	1.0778	1.0694	1.0667
70%	1.5760	1.5047	1.1124	1.0778	1.0694	1.0667
80%	1.6133	1.4483	1.1091	1.0778	1.0694	1.0667
90%	1.6270	1.3550	1.1176	1.0778	1.0694	1.0667
100%	1.6391	1.2432	1.1357	1.0778	1.0694	1.0667

**Table 2:** Complexity of voltage for the LG fault.

Tap	Fault Phase A	Non-Faulty Phase B	Non-Faulty Phase C	Normal Phase A	Normal Phase B	Normal Phase C
10%	0.268	1.4729	1.4733	1.0842	1.0615	1.0612
20%	0.2679	1.4863	1.4874	1.0842	1.0615	1.0612
30%	0.268	1.501	1.5027	1.0842	1.0615	1.0612
40%	0.2679	1.5165	1.5193	1.0842	1.0615	1.0612
50%	0.268	1.5336	1.5373	1.0842	1.0615	1.0612
60%	0.2679	1.5525	1.557	1.0842	1.0615	1.0612
70%	0.2679	1.5733	1.5789	1.0842	1.0615	1.0612
80%	0.268	1.5857	1.5966	1.0842	1.0615	1.0612
90%	0.268	1.4428	1.455	1.0842	1.0615	1.0612
100%	0.2681	1.1942	1.2036	1.0842	1.0615	1.0612

**Table 3:** Complexity of current for the TT fault at phase A of fault phase.

	Tap#B	2.1	2.2	2.3	2.4	2.5	2.6	2.7	2.8	2.9	2
Tap#A											
2		1.4467	1.4382	1.4314	1.4258	1.418	1.4116	1.4067	1.4031	1.4005	1.3987
2.1			1.4686	1.4581	1.4494	1.4424	1.4334	1.4259	1.42	1.4154	1.412
2.2				1.4891	1.4769	1.4669	1.4584	1.4482	1.4398	1.433	1.4276
2.3					1.5087	1.4947	1.4832	1.4737	1.4626	1.4532	1.4456
2.4						1.5293	1.5119	1.499	1.4883	1.4756	1.4663
2.5							1.5514	1.5303	1.5142	1.5024	1.4897
2.6								1.5743	1.5497	1.5311	1.5162
2.7									1.5938	1.5695	1.5483
2.8										1.4878	1.5901
2.9											1.2421
Normal		1.0778	1.0778	1.0778	1.0778	1.0778	1.0778	1.0778	1.0778	1.0778	1.0778

**Table 4:** Complexity of voltage for the TT fault at phase A of fault phase.

	Tap#B	2.1	2.2	2.3	2.4	2.5	2.6	2.7	2.8	2.9	2
Tap#A											
2		0.2766	0.268	0.268	0.268	0.268	0.268	0.268	0.268	0.268	0.268
2.1			0.6533	0.5453	0.4465	0.3579	0.2791	0.2644	0.2644	0.2643	0.2643
2.2				1.0128	0.9503	0.8967	0.8459	0.7967	0.7481	0.6984	0.6434
2.3					1.1724	1.1176	1.071	1.0282	0.9881	0.9502	0.9141
2.4						1.276	1.2258	1.1825	1.1427	1.1057	1.0711
2.5							1.3526	1.3045	1.2635	1.2258	1.1909
2.6								1.4138	1.3661	1.3267	1.2906
2.7									1.4699	1.4187	1.3785
2.8										1.5241	1.4571
2.9											1.5703
Normal		1.0842	1.0842	1.0842	1.0842	1.0842	1.0842	1.0842	1.0842	1.0842	1.0842

**Table 5:** Complexity of current for the LG fault.

Distance	Fault Phase A	Non-Faulty Phase B	Non-Faulty Phase C	Normal Phase A	Normal Phase B	Normal Phase C
0 km	1.4236	1.544	1.4808	1.0778	1.0694	1.0667
0.5 km	1.4243	1.545	1.4819	1.0778	1.0694	1.0667
1 km	1.425	1.546	1.483	1.0778	1.0694	1.0667
2 km	1.4263	1.5481	1.485	1.0778	1.0694	1.0667
4 km	1.4291	1.5522	1.4891	1.0778	1.0694	1.0667
6 km	1.4319	1.5563	1.4927	1.0778	1.0694	1.0667
8 km	1.4347	1.5607	1.4954	1.0778	1.0694	1.0667
10 km	1.4376	1.5651	1.4977	1.0778	1.0694	1.0667
12 km	1.4405	1.5696	1.4994	1.0778	1.0694	1.0667
14 km	1.4435	1.5743	1.5008	1.0778	1.0694	1.0667
16 km	1.4464	1.5791	1.5017	1.0778	1.0694	1.0667
18 km	1.4493	1.583	1.5024	1.0778	1.0694	1.0667
20 km	1.4524	1.5853	1.5027	1.0778	1.0694	1.0667

**Table 6:** Complexity of voltage for the LG fault.

Distance	Fault Phase A	Non-Faulty Phase B	Non-Faulty Phase C	Normal Phase A	Normal Phase B	Normal Phase C
0 km	0.2574	1.4607	1.4604	1.0842	1.0615	1.0612
0.5 km	0.2573	1.4619	1.4604	1.0842	1.0615	1.0612
1 km	0.2572	1.4632	1.4604	1.0842	1.0615	1.0612
2 km	0.257	1.4658	1.4605	1.0842	1.0615	1.0612
4 km	0.2617	1.471	1.4608	1.0842	1.0615	1.0612
6 km	0.5707	1.4762	1.4611	1.0842	1.0615	1.0612
8 km	0.7652	1.4816	1.4616	1.0842	1.0615	1.0612
10 km	0.8758	1.4872	1.4621	1.0842	1.0615	1.0612
12 km	0.9542	1.4929	1.4627	1.0842	1.0615	1.0612
14 km	1.0162	1.4987	1.4635	1.0842	1.0615	1.0612
16 km	1.0671	1.5046	1.4643	1.0842	1.0615	1.0612
18 km	1.1101	1.5106	1.4651	1.0842	1.0615	1.0612
20 km	1.1473	1.5167	1.466	1.0842	1.0615	1.0612

## Data Availability

Data are contained within the article.
